# Dollars and Ideas

**Published:** 1861-10

**Authors:** 


### DOLLARS AND IDEAS.

A DOLLAR buys the poor man a dinner, and he passes it off. Tomorrow the same dollar does the same thing for some other man, and so it goes on, passing from hand to hand, feeding multitudes; and, so far from growing less in value, it is brightened by the handling, and, at the end of months and years of service, is worth as much as ever, while its very glistening makes it more tempting than one blackened by age and disuse.

"Every new idea is worth a silver dollar, young gentlemen," was a favorite stimulus to study, with our loved and venerated teacher, Ebenezer Sharp, long since in his Christian grave, and whose memory we bless.

Later on, we elsewhere learned, that there was a striking and practical resemblance between good ideas and good dollars; both grew more shining by use, for rust corrupted not, and disuse did not canker them away. Genuine dollars, like genuine ideas, increase the sum total, by circulation, by being passed around. Let us drop the dollar, and take up the idea. Truth is used by conversation and writing, and the oftener it is used thus, the brighter does it become, the more readily can it be applied, and the wider will be the range of uses which can be made of it. That which makes all the difference between the empty head and the crowded one, between a full man and a fool, is the facility with which things known can be made use of. It is not the mere fact of having money on hand, that makes a successful operator in Wall-street ; it is the having it at command at an hour's notice, with the frequent judicious use of it : and that very use gives more power.

We take up a dollar, coined in the infancy of our Republic ; the man who moulded it has been dead for half a century. And yet, how many throbs of gratification has it excited, as it has been laid in the hand ; and how many a hungry mouth has it fed ; and yet it is as good as ever, and may continue to gladden the joyless, and feed the hungry, for a hundred years to come. But there is a remarkable difference between a good idea and a good dollar. The dollar is always received with pleasure ; but it is parted with, with a pang. But a good idea, well coined in words, gratifies him who gives, as well as him who receives.

One sterling truth, clothed in words of vigor, keenness, or sweetness, spoken or printed, passes from mouth to mouth, from paper to paper, from page to page, and will happify a million hearts. The dollar can be used by only one at a time, and but at one place in the same instant ; but the mental coin, by the art of all arts, can be used by multitudes, in all lands, at the same instant, distilling its sweetness on both giver and receiver everywhere, to be perpetuated for generations yet unborn.

The responsibilities of the press, of a writer, how amazing, how imperative, in the great contest of ages, between ignorance and error ! And the privilege of an ability to clothe our ideas in words that burn or bless ; in resistless power, or in honied sweetness, to wake up the sleepy and the lazy, or help up the weary, the lonely, or desponding, how high, how dear ! Men of Israel ! men of mind ! help ! These fields are open to you. Use them with power, with love ; and lay out yourselves to coin a moral dollar once a month—clear, bright, beautiful, well defined—which shall instruct or gladden many a living heart, and continue to do the same, long after "the harvest is ended" for you, and the clods have rested on your grave. Do it now, and do it till you die, for "The night cometh when no man can work."

